# Site-specific programming of the host epithelial transcriptome by the gut microbiota

**DOI:** 10.1186/s13059-015-0614-4

**Published:** 2015-03-28

**Authors:** Felix Sommer, Intawat Nookaew, Nina Sommer, Per Fogelstrand, Fredrik Bäckhed

**Affiliations:** The Wallenberg Laboratory and Sahlgrenska Center for Cardiovascular and Metabolic Research, Department of Molecular and Clinical Medicine, University of Gothenburg, Gothenburg, 41345 Sweden; Department of Chemical and Biological Engineering, Chalmers University of Technology, Gothenburg, 41296 Sweden; Present Address: Comparative Genomics Group, Biosciences Division, Oak Ridge National Laboratory, Oak Ridge, TN 37831 USA; Novo Nordisk Foundation Center for Basic Metabolic Research, Section for Metabolic Receptology and Enteroendocrinology, Faculty of Health Sciences, University of Copenhagen, Copenhagen, DK-2200 Denmark

## Abstract

**Background:**

The intestinal epithelium separates us from the microbiota but also interacts with it and thus affects host immune status and physiology. Previous studies investigated microbiota-induced responses in the gut using intact tissues or unfractionated epithelial cells, thereby limiting conclusions about regional differences in the epithelium. Here, we sought to investigate microbiota-induced transcriptional responses in specific fractions of intestinal epithelial cells. To this end, we used microarray analysis of laser capture microdissection (LCM)-harvested ileal and colonic tip and crypt epithelial fractions from germ-free and conventionally raised mice and from mice during the time course of colonization.

**Results:**

We found that about 10% of the host’s transcriptome was microbially regulated, mainly including genes annotated with functions in immunity, cell proliferation, and metabolism. The microbial impact on host gene expression was highly site specific, as epithelial responses to the microbiota differed between cell fractions. Specific transcriptional regulators were enriched in each fraction. In general, the gut microbiota induced a more rapid response in the colon than in the ileum.

**Conclusions:**

Our study indicates that the microbiota engage different regulatory networks to alter host gene expression in a particular niche. Understanding host-microbiota interactions on a cellular level may facilitate signaling pathways that contribute to health and disease and thus provide new therapeutic strategies.

**Electronic supplementary material:**

The online version of this article (doi:10.1186/s13059-015-0614-4) contains supplementary material, which is available to authorized users.

## Background

The human gut harbors a diverse microbial community (gut microbiota) that mainly consists of bacteria. Their combined genomes (the microbiome) encode more than 10 million genes, outnumbering our own genetic potential by two to three orders of magnitude [1-3], and provide biochemical and metabolic functions that complement our physiology. For example, the gut microbiota metabolizes otherwise indigestible polysaccharides and produces essential vitamins, instructs the development of the intestinal epithelium and immune system, and plays a key role in maintaining tissue homeostasis [4]. Maintaining symbiosis seems to be a key requirement for health as dysbiosis is associated with the development of common diseases, including obesity, atherosclerosis and inflammatory bowel disease [5-7].

Within the gastrointestinal tract, microbial distribution varies considerably and diversity increases along the length of both the gut and the tissue-lumen axis [8], impacting function [9] and architecture [10] of the gut. A few recent studies have investigated microbiota-induced host responses along the length of the gut [11-15]. However, these reports used intact tissue samples and could not, therefore, be used to draw conclusions about the cellular responses in the gut epithelium since intestinal epithelial cell (IEC) types are differentially distributed in the intestinal tissue. The epithelium tips mainly contain differentiated epithelial cells, such as enterocytes and goblet cells, whereas the crypts mainly consist of proliferative and Paneth cells. Thus, the resident microbiota might induce alternative local host responses in different cell populations within the intestinal epithelium, and along the crypt-villus axis, which may contribute to different functional properties of the tissue.

To investigate the microbiota-induced transcriptional responses in specific and well-defined cell populations of the host’s epithelium, we used extensive microarray analysis of laser capture microdissection (LCM)-harvested ileal and colonic tip and crypt fractions from germ-free (GF) and conventionally raised (CR) mice as well as during the time course of colonization of GF mice.

## Results

### The gut microbiota induces specific differential transcriptional responses depending on location in the intestinal epithelium

**Figure 1:**
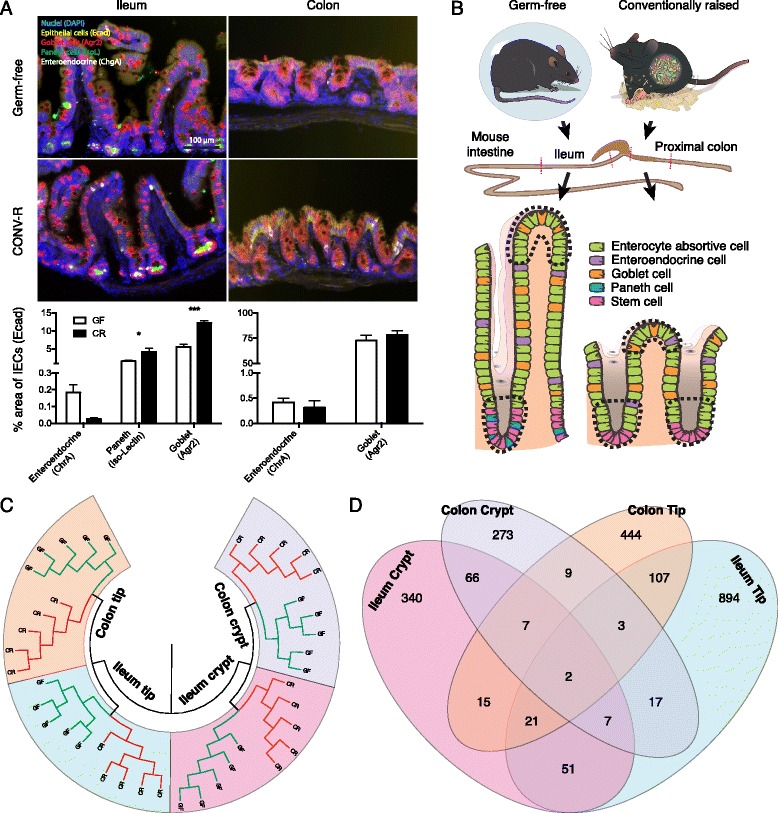
The microbiota elicits specific transcriptional responses in different locations of the gut epithelium. (**A**) Immunostaining using epithelial cell type-specific antibodies and quantification. n = 8 (GF) and 9 (CR); *P < 0.05, ***P < 0.001 (two-way ANOVA). All pictures are in the same magnification. The scale bar indicates 100 μm. Data show mean ± standard error of the mean. (**B**) Schematic overview of the experimental setup. Tip and crypt epithelial fractions were harvested by LCM from ileum and colon of GF and CR mice and then used for microarray analysis. (**C**) Hierarchical clustering of the microarray data. (**D**) Venn diagram of microbiota-dependent genes (*P* < 0.001 for GF versus CR) in the four different LCM fractions.

To investigate a potential effect of the gut microbiota on IEC differentiation, we determined the cellular composition in ileum and proximal colon of GF and CR mice using immunostaining with a recently developed multi-channel setup [16] and an automated slide scanning and picture analysis pipeline. Overall, we found only minor alterations of IEC type abundance between GF and CR mice with slightly more Paneth and goblet cells in CR ileum (Figure 1A).

As the gut microbiota only had a minor impact on IEC composition we investigated microbiota-induced responses in defined IEC populations using LCM to isolate tip and crypt fractions from the ileum and proximal colon of GF and CR mice (Figure 1B). We performed 40 microarray hybridizations using the LCM-harvested tip and crypt epithelial fractions to evaluate their transcriptional profiles according to bacterial status and location. Hierarchical clustering revealed a clear separation of the samples depending on epithelial population (tip versus crypt), then according to tissue (ileum versus colon) and finally according to bacterial status (GF versus CR) (Figure 1C).

**Figure 2:**
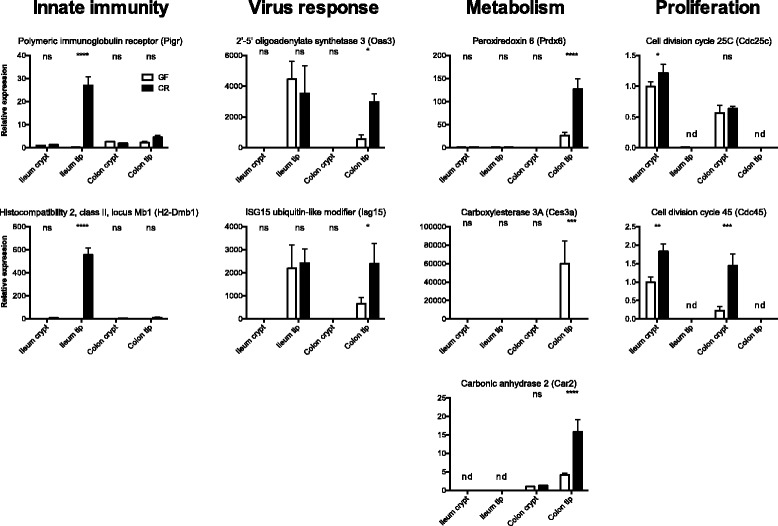
Quantitative PCR validation of site-specific microbiota-induced transcriptional responses in the gut epithelium. Tip and crypt epithelial fractions were harvested via LCM from ileum and colon of GF and CR mice. RNA was isolated from each fraction, linear amplified and directly used for quantitative PCR analysis. n = 4 to 5 per group. Data show mean ± standard error of the mean. *P < 0.05, ***P < 0.001, ****P < 0.0001 (two-way ANOVA). ns, not significant; nd, not detectable.

We used statistical Student *t*’s test scoring for bacterial status to identify microbiota-regulated genes. In total, 2,256 genes (*P* < 0.001) were regulated by the gut microbiota, most of them in the ileum tip (Additional file 1). Eighty-six percent of all microbiota-regulated genes were specific for the individual sample types (ileum crypt, 340; ileum tip, 894; colon crypt, 273; colon tip, 444; Figure 1D), indicating highly site-specific transcriptional responses of the intestinal epithelium to the microbiota. The site-specific microbial modulation of host gene expression was validated for several representative genes of different functional categories using quantitative PCR (Figure 2).

### Functional alterations in the intestinal epithelium caused by the microbiota

**Figure 3:**
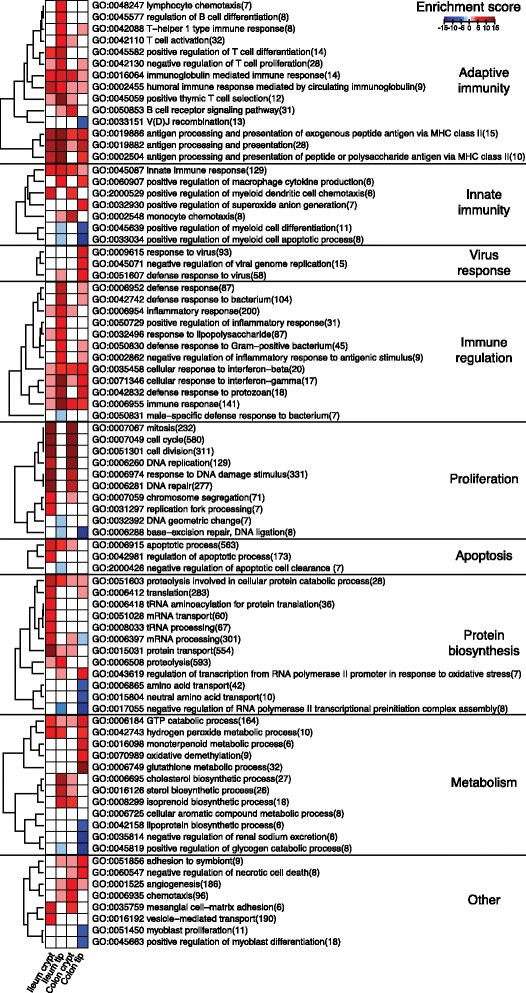
Microbiota-responsive gene functions in different locations of the gut epithelium. Gene ontology analysis of microbiota-dependent genes in the LCM fractions. Only terms with *P* < 10^−5^ in any of the four epithelial fractions and more than five members were considered. Figure depicts log-transformed adjusted *P*-values for GF versus CR comparison.

We next investigated functions of the microbially regulated genes in the four epithelial fractions using gene ontology (GO) analysis. In total, 81 GO categories were altered significantly (*P* < 10^−5^ with more than 5 members; Figure 3; see Additional file 2 for full list), with most GO categories being enriched in the presence of the microbiota. As expected, about half of all GO terms were related to immune functions and were predominantly increased in the tip fractions of both ileum and colon. Antigen presentation and interferon-γ responses were enriched in both tissues. In contrast, processes related to T-cell regulation were restricted to the ileum tip whereas virus response functions were mainly enriched in the colon tip. Several GO terms related to the cell cycle were highly enriched in the crypts of both ileum and colon, indicating altered cell proliferation in the presence of the gut microbiota. Increased proliferation in crypts from CR compared with GF mice was confirmed by staining sections from ileum and crypt with the proliferative marker Ki-67 (Additional file 3), which is in agreement with previous publications [4,14,17]. In addition, several functions related to protein biosynthesis were enriched in the crypts. Metabolic processes were also altered predominantly in the tips of both ileum and colon by the gut microbiota. However, the specific functions differed in the two tissues. In the ileum tip cholesterol/lipid metabolism was enriched whereas in the colon tip glutathione redox processes and production of antibacterial monoterpenoids were enriched. By analyzing the glutathione-S-transferase activity in isolated tip epithelium from ileum and colon of GF and CR mice, we validated that glutathione-S-transferase activity was increased by the microbiota in colonic, but not ileal, tip epithelium (Additional file 4). The only GO terms that were depleted in the host epithelium by the presence of the microbiota were amino acid transport, sodium excretion and glycogen catabolism in the colon tip.

### The gut microbiota engages distinct transcriptional regulators in the different host epithelial fractions

**Figure 4:**
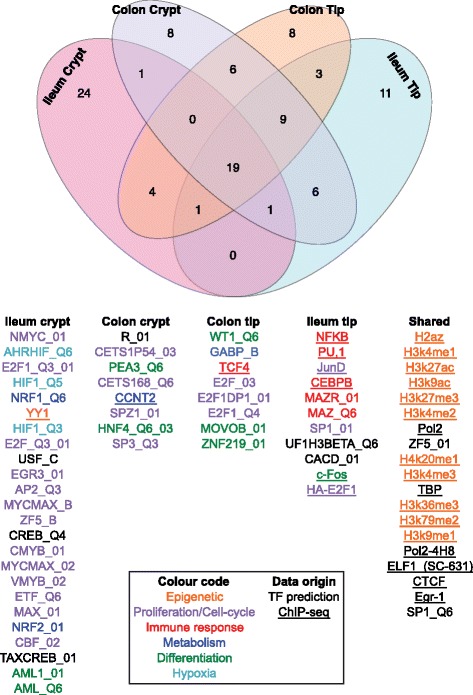
Regulatory factors enriched among promoters of microbiota-responsive genes. Transcription factor binding sites were predicted in promoter sequences (1 kb) of microbiota-responsive genes. Lists of microbially responsive genes were also compared with target genes inferred from published ChIP-seq data. Results were merged and only the 50 most significantly enriched regulators considered for each of the four epithelial fractions. TF, transcription factor.

Next, we used two different approaches to identify regulatory pathways through which the gut microbiota elicits the observed transcriptional responses in the epithelium: (i) we screened the promoter sequences (1 kb) of all microbiota-altered genes for transcription factor binding sites; and (ii) we compared the lists of microbially responsive genes with target genes of transcriptional regulators using published chromatin immunoprecipitation (ChIP)-seq datasets (Additional file 5). We then considered only the 50 most significantly enriched regulators for each of the four epithelial fractions, yielding a total of 101 unique factors. This analysis revealed that 19% (19 of 101) of the enriched regulatory factors were shared between the four epithelial fractions (Figure 4). Most of the shared regulators represented global factors such as histone modifications, which could account for the broad microbially induced alterations in the host transcriptome (10% of all genes). About 50% (51 of 101) of the predicted regulatory factors were specifically enriched in one of the epithelial fractions and mainly associated with cellular processes that were also enriched in GO analysis. In the ileum crypt, 13 of the 24 enriched regulatory factors were associated with cell cycle control and proliferation. These included, for example, the transcription factors E2F1 (E2F transcription factor 1) and the heterodimer MYC/MAX (v-myc avian myelocytomatosis viral oncogene homolog/MYC associated factor X), whose target genes *Cdc25c* (cell division cycle 25C), *Cdc6*, *Aurka* (aurora kinase A), *Nbn* (nibrin) and *Cep55* (centrosomal protein 55) [18-24] are all involved in different aspects of cell cycle control and were among the most significantly altered genes in ileum crypt. Similarly, in the ileum tip, 5 of the 11 enriched factors were associated with immune processes and included, for example, the transcription factors NFkB (nuclear factor-kappaB) and CEBPB (CCAAT/enhancer-binding protein beta). Their target genes *H2-DMb1* (histocompatibility 2, class II, locus Mb1), *Pigr* (polymeric immunoglobulin receptor) or *Mif* (macrophage migration inhibitory factor) [25-31] were among the most significantly altered genes in ileum tip and are involved in immune regulation: antigen presentation, IgA secretion and macrophage chemotaxis. CEBPB seemed to have a dual role in the ileum tip, regulating not only immune but also several metabolic genes, including, for example, the glycolysis genes *Pfkfb3* (6-phosphofructo-2-kinase/fructose-2,6-biphosphatase 3) and *G6pc* (glucose-6-phosphatase) as well as the gut hormone *Fgf15* (fibroblast growth factor 15) [32-36], thereby integrating immune and metabolic responses.

### Kinetics of the transcriptional programming by the gut microbiota

**Figure 5:**
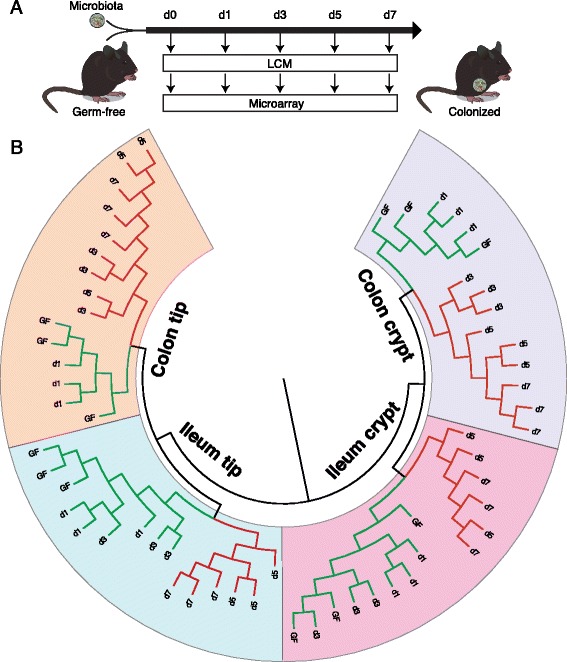
The colonic epithelium responds quicker to microbial exposure than the ileal. (**A**) Schematic overview of the experimental setup. GF mice were colonized with a normal microbiota. Tip and crypt epithelial fractions were harvested by LCM on days (d) 0, 1, 3, 5 and 7 during the time course of colonization and then used for microarray analysis. (**B**) Hierarchical clustering of the microarray data.

To elucidate the temporal sequence of the transcriptional responses elicited by the microbiota in the different epithelial fractions, we colonized GF mice with a normal microbiota and performed LCM followed by microarray as above using samples harvested before and 1, 3, 5 and 7 days after colonization (Figure 5A). Again hierarchical clustering clearly separated the samples depending on epithelial fraction, then tissue and finally bacterial status (Figure 5B). On the level of bacterial status, the samples clustered in different groups depending on the tissue. Ileal samples GF, d1 (day 1 post-colonization) and d3 clustered against d5 and d7 whereas colonic samples GF and d1 clustered against d3, d5 and d7. Not all but a greater proportion of the genes responded faster and to a full CR level to the microbial colonization in colon than in ileum (Additional files 1 and Additional file 6). Together, this suggests that the transcriptional response of the epithelium seems faster in the colon than in the ileum.

**Figure 6:**
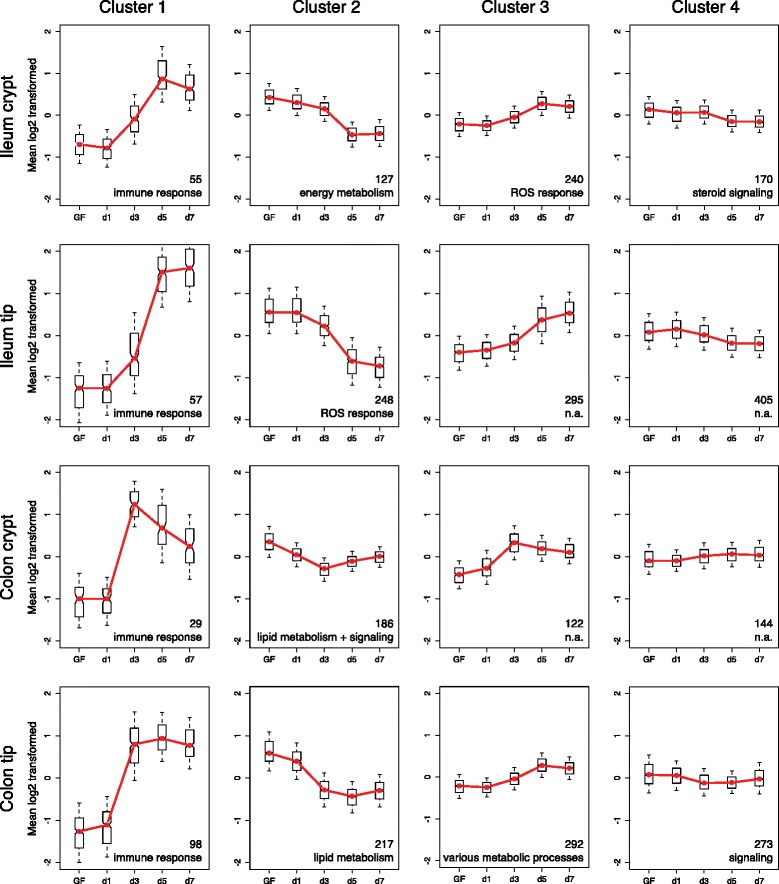
Clustering of microbially altered genes according to their responses during colonization of GF mice. For each LCM fraction, normalized gene expression values were analyzed for their changes over time during colonization. Those genes with significant changes between at least two time points were clustered into four different clusters solely according to their expression profile using a consensus-clustering approach. Besides their expression pattern, clusters of different tissue fractions are completely unrelated (for example, cluster 1 of ileum crypt or tip). Data show mean ± standard deviation. Numbers indicate how many genes are contained in the respective cluster. The noted function refers to the most abundant GO term associated with the clustered genes.

We then clustered all genes according to their expression variation over the colonization period (Figure 6). Clustering yielded four different clusters for each of the analyzed epithelial fractions (Additional file 7). Cluster 1 contained genes whose expression increased drastically between days three and five in the ileum or days one and three in the colon. This cluster mainly included genes related to immune functions. However, out of the 239 genes assigned to cluster 1 for all epithelial fractions, very few were shared whereas about 55% (133 out of 239) were specific for one of the four epithelial fractions. Most cluster 1 genes specific for ileum crypt were associated with antigen sampling and pattern recognition receptor signaling and included *H2-Ab1* (histocompatibility 2, class II antigen A, beta 1), *Cd14* (CD14 antigen) and *Tifa* (TRAF-interacting protein with forkhead-associated domain). Most ileum tip-specific cluster 1 genes had functions in antigen sampling and mucosa fortification; for example, *Pigr*, *Nos2* (inducible nitric oxide synthase 2), *Lbp* (lipopolysaccharide binding protein) and *Sprr2a* (small proline-rich protein 2A). The cluster 1 colon crypt-specific genes were mainly chemokines such as *Cxcl9* (chemokine (C-X-C motif) ligand 9), *Cxcl10* or *Cxcl11*. Finally, most cluster 1 genes specific for the colon tip were interferon-dependent, such as *Ifi204* (interferon activated gene 204), *Ifit1* (interferon-induced protein with tetratricopeptide repeats 1) and *Irf8* (interferon regulatory factor 8).

The genes of cluster 2 showed a decrease in expression over time. Cluster 2 mainly contained genes with metabolic functions, namely lipid metabolism, but as for cluster 1, most cluster 2 genes were specific for a single epithelial fraction. The lipid metabolism cluster 2 genes in the ileum crypt included *Cryl1* (crystallin, lambda 1), *Hmgcs2* (3-hydroxy-3-methylglutaryl-coenzyme A synthase 2) and *Hsd3b3* (hydroxy-delta-5-steroid dehydrogenase, 3 beta- and steroid delta-isomerase 3) whereas those in the colon crypt included *Kdsr* (3-ketodihydrosphingosine reductase), *Hsd17b4* (hydroxysteroid (17-beta) dehydrogenase 4), *Hadh* (hydroxyacyl-coenzyme A dehydrogenase) and *Ces1d* (carboxylesterase 1D) and those in the colon tip included *Apob* (apolipoprotein B) or *Apoa1*. In contrast, most cluster 2 genes specific for the ileum tip were involved with responses to reactive oxygen such as *Sod1* (superoxide dismutase 1, soluble), *Gstp1* (glutathione S-transferase, pi 1) and *Cat* (catalase).

The remaining two clusters, 3 and 4, included genes with expression patterns similar to those of clusters 1 and 2, respectively, but with smaller changes in their expression levels. Clusters 3 and 4 contained a higher number of genes and GO analysis did not yield dominant cellular processes (Additional file 8). However, as for clusters 1 and 2, most genes of clusters 3 and 4 were also specific for an epithelial fraction. Together, this demonstrates the site-specificity of the microbiota-induced transcriptional responses.

Next, we screened for putative transcriptional regulatory factors that are enriched among promoter sequences of the microbially regulated gene clusters (Additional file 9). Of the 50 most significant regulatory factors, 63 to 69% were specific for one of the four clusters in any epithelial fraction. Furthermore, only about 20% of the regulatory factors in clusters 1 or 2 of the four epithelial fractions were shared, although they all contained genes with immune or metabolic functions, respectively. This indicates, therefore, that the gut microbiota elicits its site-specific transcriptional responses in the different host epithelial fractions using distinct regulatory networks.

## Discussion

We systematically investigated the microbiota-induced transcriptional responses in specific subpopulations of intestinal epithelial cells using extensive microarray analysis of LCM-harvested ileal and colonic tip and crypt fractions from GF and CR mice as well as during the time course of colonization. This analysis revealed that approximately 10% of the host’s transcriptome was microbially regulated and that the transcriptional response of the epithelium towards the microbiota was faster in the colon than in the ileum. The main gene functions that were affected by the microbiota were host immunity, cell proliferation and metabolism. However, the microbial impact on epithelial gene expression was also highly site specific as the microbiota-induced host transcriptional responses differed not only between ileal and colonic samples but also among tip and crypt fractions. For example, we corroborated that the gut microbiota promoted cell proliferation in crypts of the ileum and colon as well as glutathione-S-transferase activity in the colonic tip epithelium. The microbiota seemed to use different networks of transcriptional regulators to elicit the observed specific alterations in the epithelial transcriptome profiles. To facilitate a further deciphering of the microbiota-epithelium interactions in the intestine by the research community we generated an online database [37] using the data presented here which allows easy access to our dataset presented here.

### Most microbially induced transcriptional changes are restricted to epithelial cells

In a previous study we compared the transcriptional profiles in duodenum, jejunum, ileum and colon between GF and CR mice [15]. Few other recent studies also investigated the instructive capacity of the gut microbiota on the host transcriptome in a systematic way during a non-diseased state. El Aidy and colleagues explored the interaction between the microbiota and the host intestinal tissue during colonization of germ-free mice in jejunum, ileum and colon [11,13,14]. Furthermore, a few recent studies analyzed a microbial impact on isolated but unfractionated epithelium [38-40]. In our current study we extended these earlier findings by using LCM-harvested epithelial fractions instead of whole tissue samples or unfractionated epithelium, thus enabling the analysis of the interactions between the gut microbiota and specific epithelial fractions. The value of our experimental approach is demonstrated by the fact that many findings of microbially regulated genes or processes observed in intact tissues or even in unfractionated epithelium from previous studies are not universal but limited to specific epithelial fractions. For example, Donohue and colleagues [38] showed that the microbiota alters colonocyte energy metabolism by providing butyrate as energy substrate and thus upregulating genes involved in ß-oxidation (*Acads*, *Hadha*) or the tricarboxylic acid cycle (*Aco2*, *Mdh2*). However, we found that the changes in expression of these genes were restricted to IECs of the colon tip epithelium. Pott and colleagues [39] showed that the microbiota modulates expression of *Tlr4* in the small intestinal epithelium. Our data demonstrate that this effect is restricted to the ileum tip. Camp and colleagues [40] showed similar epithelial responses in ileum and colon towards the microbiota. However, we found that many of the microbially induced functional alterations are site-specific: immune responses in the tip epithelium of ileum and colon, regulation of cell proliferation in the crypt fractions of ileum and colon, whereas lipid metabolism and glutathione metabolism were specific for ileum and colon tip fractions, respectively. In a recent study, Kim and colleagues [41] reported transcriptional profiles for Lgr5-positive stem cells, IEC progenitors of the secretory and enterocyte lineage, and mature enterocytes. To explore whether the microbiota-induced transcriptional changes reported here were partly due to alterations in abundance or activity of specific IEC types, we aimed to compare these two epithelial transcriptome datasets. Unfortunately, these comparisons were technically inappropriate due to confounding factors between the two datasets, for example, the use of different microarray technologies and normalization of the data.

In line with the data from previous reports, we found that the gut microbiota has wide-ranging effects on host gene expression, mainly inducing genes with immune functions in the ileum and genes with metabolic function in the colon. In fact, most genes, for example *Cxcl5*, *Cxcl9* or *Sprr1a*, which function in mucosa fortification, showed a similar expression pattern as reported in our previous study [15]. This might, therefore, indicate that many of the microbiota-induced transcriptional changes are based in epithelial cells of the intestinal mucosa. However, in contrast to the earlier reports we did not detect alterations in several immune genes previously found to be microbially regulated. For example, we found no increase and in fact not even detectable levels of the regulatory T cell markers *T-bet* and *Foxp3* or the Th17 gene *Cxcl-17* during conventionalization as was reported previously [14,42]. These differences are most likely due to our experimental design involving LCM, which allowed analyzing the transcriptional responses of isolated epithelial fractions and therefore did not contain regulatory T cell or Th17 lymphocytes, demonstrating that many of the microbially induced immune responses detected by studies using whole tissues might be due to the presence of immune cells. Thus, restricting the study of microbially induced expression to epithelial cells, as we performed here, allowed delineation towards only this cell type. However, this experimental design also represents a potential limitation as possible interactive or indirect responses - for example, together with or via sensory immune cells - were omitted from this analysis due to its epithelial focus. We can thus not exclude that some genes are indirectly regulated by the gut microbiota through its interactions with immune cells. Furthermore, our analyses do not allow us to identify which epithelial cells respond to the gut microbiota. This will require further experiments using single cell analyses.

### Epithelial cells in different locations respond differentially to the microbiota

The use of LCM facilitated a detailed analysis of host-microbial interactions in specific subpopulations of intestinal epithelial cells and revealed that the majority of microbially regulated genes (1,941 of 2,256) were specific for one of the four epithelial fractions (ileum crypt, ileum, tip, colon crypt and colon tip). For example, we found the gene *Angptl4*, which is involved in lipid metabolism and was already described earlier as microbially regulated [43], to be repressed by bacteria specifically in the ileal tip epithelium (*P* < 0.001). Similarly, the microbially regulated genes *Fgf15* and *Sprr1a* [15,44] were only modulated in the ileal and colonic tip epithelium, respectively.

Since the gut microbiota only had a minor impact on IEC composition as supported by published data [14,45], the observed site-specific microbial impact on epithelial gene expression seems to be mainly caused by direct transcriptional reprogramming in the epithelial fractions. There are several factors that could account for the strong site specificity. First, the tip and crypt fractions contain different types of epithelial cells (enterocytes and goblet cells in the tip versus stem and Paneth cells in the crypt) with different cellular functions. Second, the microbial composition differs among both the proximal-distal and tissue lumen axes [4]. Thus, microbiota-derived signaling stimuli likely differ from ileum to colon and between tip and crypt in the same intestinal segment and might thereby induce different signaling events leading to alternative transcriptional responses in the host epithelium. In line with this hypothesis, by screening for enriched transcriptional regulators among the microbially responsive genes, we identified regulatory factors that were specifically enriched in a single epithelial fraction and associated with the most significant GO functions (for example, NFkB and CEBPB) with the immune genes *Pigr* or *Mif* in the ileum tip epithelium. This is further supported by our observation that, when clustering all microbially regulated genes according to their changes in expression during colonization, we identified different genes and regulatory factors in each of the four gene clusters of a single epithelial fraction (for example, clusters 1 to 4 of ileum tip) but also among the same cluster of different epithelial fractions (for example, cluster 1 in ileum crypt, ileum tip, colon crypt and colon tip), although they often are involved in similar cellular functions such as lipid metabolism. This highlights, therefore, the specific regulatory networks engaged by the gut microbiota to alter host epithelial gene expression and function according to the requirements in that particular niche. It is important to note, however, that many of the published databases that we used to predict transcriptional regulators were not derived from mouse intestine and thus functional proof of the predicted transcriptional regulatory interactions in the intestine remain to be demonstrated.

### Microbial impact on epithelial reprogramming

The gut microbiota has been implicated to impact the host’s epigenetic program [46-49]. Bacterially produced folate is the essential methyl group donor during histone methylation and thereby leads to inhibition of transcription [49]. In contrast, the microbially produced short-chain fatty acids butyrate and propionate are potent histone deacetylase (HDAC) inhibitors and thereby increase transcriptional activity [47]. It was recently shown that epithelial HDAC3 is critical for intestinal homeostasis by proper integration of microbially derived signals [46]. Many of the GO functions that were enriched in our analysis, including lipid metabolism, glutathione metabolism, antigen presentation or immune response, were also enriched in IECs isolated from HDAC3 knockout compared with wild-type mice [46]. Furthermore, many of the transcriptional regulators that we found enriched among the microbially regulated genes were methylations and acetylations of histones, including the prototypical HDAC3 target H3k9ac. Thus, many of the microbially responsive genes might be regulated epigenetically, potentially via short-chain fatty acid-repressed HDACs or a folate-induced increase in DNA methylation.

### The colonic epithelium appears to respond faster to microbial stimuli than the ileal

Based on these associations between the gut microbiota and host epigenetics, we investigated whether the gut microbiota can impact epithelial reprogramming. Therefore, we analyzed the kinetics in the epithelial response to microbial colonization expecting a delayed instead of immediate responses in the tip compared with the crypt fractions since all epithelial cells originate from the crypts. In contrast to our hypothesis we did not note any delayed responses in the tips compared with crypts but we found that, on average, the colonic epithelium appeared to respond faster than the ileal epithelium. The faster response in the colonic versus the ileal tip fraction might be explained by a shorter distance between the proliferative stem cells in the crypt and the differentiated cells in the tip leading to a faster epithelial turnover time: 1 to 2 days versus 3 to 5 days in the colon versus ileum, respectively [50,51]. This, however, would not explain why the crypts in the colon also responded faster to microbial colonization than ileal crypts. This is particularly intriguing as the colon is more protected against bacterial contact than the ileum owing to a thicker and continuous mucus layer [52]. On the other hand, the colon harbors the highest density of bacteria and therefore potentially more signals to induce faster transcriptional responses [8]. Another possible explanation for the faster response in the colon might be that the microbiota used for colonization of the mice was harvested from the colon of donor mice, and thus the colonizing microbiota might be more adapted to the colonic environment. Furthermore, apart from direct transcriptional control, other processes such as mRNA stability or IEC turnover and differentiation might also contribute to the overall transcript levels.

## Conclusion

Our data show that the gut microbiota induces wide-ranging host responses. Epithelial cells are the primary responders to microbial signals but not all epithelial cells respond equally to microbial contact. In fact, the microbiota-induced responses are extremely site specific. Therefore, the database presented here will facilitate deciphering the microbiota-epithelium interactions in the intestine.

## Materials and methods

### Mice

C57Bl6/J female 12-week-old mice were maintained under standard specific pathogen free or GF conditions as described previously [53]. Mice were kept under a 12-h light cycle and fed autoclaved chow diet *ad libitum* (Labdiet, St Louis, MO, USA). For colonization the cecal content from an adult CR mouse was resuspended in 5 ml sterile reduced phosphate-buffered saline (PBS) and GF mice were orally gavaged with 200 μl. The colonized mice were maintained in standard makrolon cages for up to seven days. Mice were killed by cervical dislocation and the intestine removed. The distal part of the small intestine (ileum) and the proximal part of the colon were excised, flushed with PBS and finally flushed and embedded with OCT freezing medium. The complete procedure took less than 5 minutes to preserve RNA integrity. Animal protocols were approved by the Gothenburg Animal Ethics Committee.

### Intestinal epithelial cell composition

Intestinal specimens were harvested and fixed in 4% paraformaldehyde prior to cryoprotection, OCT embedding and cryosectioning. Sections (10 μm) were stained using various IEC subtype-specific antibodies or lectins: rat-anti-E-cadherin (all IECs; ECCD-2, Takara, #M108, Mountain View, CA, USA), goat-anti-Anterior Gradient 2 (goblet cells; Santa Cruz, #sc54561, Dallas, Texas, USA), rabbit-anti-Chromogranin A (enteroendocrine cells; ImmunoStar, #20086, Hudson, WI, USA), Ulex europaeus lectin-FITC (Paneth cells; Sigma-Aldrich, #L9006, St. Louis, MO, USA). Secondary antibodies were donkey-anti-rat-AF594 (Life Technologies, #A21209 Carlsbad, CA, USA), donkey-anti-goat-Cy3 (Jackson Immuno Research, #705-166-147, West Grove, PA, USA) and donkey-anti-rabbit-BV421 (Nordic BioSite, #406410, Täby, Stockholm, Sweden). HOECHST (Life Technologies, #H1399) was used as DNA counterstaining. Slides were scanned using Metafer automated slide scanner (MetaSystems, Altlussheim, Baden-Württemberg, Germany) and composite pictures analyzed using the Visiopharm Integrator System program [16]. Total area for each staining was determined for complete intestinal sections and expressed as percentage of epithelial area (E-cadherin).

### Sectioning and laser capture microdissection

All used material and solutions were RNase free. Cryosections (8 μm thick) were cut from the OCT blocks at −20°C using a microtome (Leica), placed onto PEN membrane slides (ZEISS) and immediately stained for LCM. Briefly, slides were dehydrated in absolute and 70% ethanol for 30 s, dipped into RNase-free water to remove excessive OCT and then incubated in 1% cresyl violet in a 50% ethanol step for another 30 s. Slides were partially de-stained by dipping in 70% and absolute ethanol before air-drying. Slides were stored in airtight containers at −80°C until LCM. Airtight containers were equilibrated to ambient temperature before opening. LCM was performed using a PALM Microbeam Microdissection microscope (ZEISS) and fractions were collected dry. The harvested fractions were immediately lyzed in RLT containing 1% β-mercaptoethanol and stored at −80°C.

### RNA isolation

RNA was isolated from the lysates of the LCM harvested fractions using RNeasy Micro Kit (Qiagen, Hilden, Germany). RNA concentration and quality were evaluated using capillary electrophoresis on a 2100 Bioanalyzer with RNA 6000 Pico kit (Agilent Technologies, Santa Clara, CA, USA).

### Microarray

Total RNA (1,000 pg) from each sample were used to generate amplified and biotinylated sense transcript cDNA from the entire expressed transcriptome according to the Nugen Technologies (San Carlos, CA, USA) Ovation® Pico WTA System V2 (M01224v2) and Encore Biotine Module (M01111v5). GeneChip ST Arrays (GeneChip Mouse Gene 2.0 ST Array) were hybridized for 16 h in a 45°C incubator, rotated at 60 rpm. The arrays were then washed and stained using the Fluidics Station 450 and finally scanned using the GeneChip Scanner 3000 7G according to the GeneChip Expression Wash, Stain and Scan Manual (PN 702731 rev. 3, Affymetrix, Santa Clara, CA, USA). In total, 100 arrays were hybridized with five biological replicates per group in the GF versus CR experiment and three biological replicates per group in the colonization experiment.

### Transcriptome data acquisition

The microarray CEL files were normalized by quintile method with pm-only as background correction and expression values were calculated using iterative Probe Logarithmic Intensity Error (iterPlier) with expression console software (Affymetrix). To evaluate the intrinsic variability of the transcriptome data derived from different conditions, hierarchical clustering was performed based on Pearson’s correlation distance among the samples and the results presented as a dendogram plot. The normalized gene expression values were further used for differential gene expression analysis by moderated t-test [54] for pairwise comparison over different statistical hypotheses. The statistical *P*-values were further corrected for multiple testing using the Benjamini-Hochberg method. Venn diagrams of differential gene expression derived from various comparisons were drawn at adjusted *P*-value <0.001.

### Gene expression clustering during time course of colonization

First, to compare the transcriptomes of the individual samples, hierarchical clustering was performed based on Pearson’s correlation distance among the samples and the results presented as a dendrogram plot. Then, to analyze the responses of individual genes, consensus clustering was performed. To this end, the normalized gene expression values from each of the four LCM fractions were independently evaluated by their variation over the colonization period using one-way ANOVA. The statistical *P*-values were further corrected for multiple testing by the Benjamini-Hochberg method. Only genes with an adjusted *P*-value <0.001 were considered for clustering analysis to identify the common responses of genes in each LCM fraction during the colonization period. The optimum number of clusters for each individual LCM fraction was independently evaluated using the ConsensusClusterPlus package for R [55] based on Pearson’s correlation distance.

### Enrichment analysis

The enrichment analyses were performed based on GO annotation or regulatory factors. The transcription factor binding targets were retrieved and combined from the Cscan database [56], including ChIP-Seq data [34], and the ECRbase database [57]. The microRNA targets were retrieved and combined from TargetScan database [58,59] and miRanda database [60,61]. For each analyzed group of genes, their GO, transcription factor and microRNA enrichment were evaluated for significance level by gene-set enrichment analysis using PIANO package for R [62] for GO enrichment and Fisher’s exact test comparing with background *P*-value derived from random sampling (average *P*-value of 1,000 calculations using a set of randomly selected genes with the same sample size as in the gene list) for transcription factor and microRNA enrichment. Finally, the 50 most significant results for each LCM fraction were selected for further analysis.

### Expression analysis using quantitative real-time PCR

Total RNA was isolated from LCM fractions using an RNeasy Micro kit (Qiagen) and linear amplified using an Ovation Pico WTA System V2 kit (Nugen). SYBR Green Master Mix buffer (1×; Bio-Rad, Hercules, CA, USA) was used for quantitative real-time PCR at final reaction volumes of 25 μl containing 7 ng cDNA. Gene-specific results were normalized to the ribosomal protein L32 mRNA (primer sequences can be found in Additional file 10). Assays were performed in a CFX96 Real-Time System (Bio-Rad). The reactions were analyzed with the ΔΔCT method. Statistical differences between GF and CR within the LCM fractions were analyzed by two-way ANOVA using GraphPad Prism 6.

### Staining for proliferative cells

Cryosections (10 μm thick) of paraformaldehyde-fixed ileum and colon tissue of GF and CR mice were stained for proliferative cells using rabbit-anti-Ki-67 antibody (Thermo Scientific, #RM-9106-S1, Waltham, MA, USA) and Rabbit IgG Vectastain Elite ABC kit (Vector Labs, #PK-6101, Burlingame, CA, USA). Sections were counterstained with hematoxylin/eosin. Ki-67-positive cells were counted for 10 crypts per section from eight mice per group.

### Glutathione-S-transferase activity of intestinal tip epithelium

Tip epithelium from intestinal tissue was harvested as described previously [41]. Briefly, 4 cm long ileum and colon segments were taken from 11-week-old GF (n = 4) and CR (n = 5) mice, flushed with PBS and turned inside out. Tip epithelium was scraped of using a glass slide. Glutathione-S-transferase activity in the tip fractions was measured using a Colorimetric GST Activity Assay Kit (Abcam, #ab65326, Cambridge, Cambridgeshire, UK) according to the manufacturer’s protocol. Protein concentration was determined using a BCA Protein Assay Kit (Thermo Scientific, #23227). Finally, glutathione-S-transferase activity was normalized to protein concentration.

### Data access

CEL files and normalized microarray data have been deposited at the NCBI Gene Expression Omnibus repository (accession numbers GSE51910 and GSE51911). The full dataset is also available online at [37].

## Additional files

Additional file 1: Table S1. Microbially regulated genes in ileal and colonic tip and crypt fractions. Normalized intensities from both microarray hybridization experiments for all genes in all samples together with fold change and normal as well as FDR-corrected *P*-values for GF versus CR comparison. Additional file 2: Table S2. Gene ontology categories significantly altered among microbially regulated genes of ileal and colonic tip and crypt fractions. Additional file 3: Figure S1. The microbiota induces IEC proliferation in ileal and colonic crypts. Ileal and colonic sections were stained for the proliferation marker Ki-67 and positive cells counted in the crypts. Data show mean ± standard error of the mean. *****P* < 0.0001 (Student *t*’s test). Additional file 4: Figure S2. Glutathione-S-transferase activity is induced by the microbiota specifically in the tip epithelium in colon but not in ileum. Tip epithelium was isolated from ileum and colon of GF and CR mice and glutathione-S-transferase activity measured. Activity was normalized per milligram total protein. Data show mean ± standard error of the mean. **P* < 0.05 (two-way ANOVA). Additional file 5: Table S3. Regulatory factors enriched in microbially regulated genes of ileal and colonic tip and crypt fractions. Additional file 6: Figure S3. In colon a proportion of genes respond faster to microbial colonization than in ileum. Candidate gene expression in ileum crypt/tip and colon crypt/tip during colonization of GF mice with a normal microbiota. *Slfn2* (schlafen 2), *Zhx2* (zinc fingers and homeoboxes 2), *Ephx2* (epoxide hydrolase 2), *Ifi44* (interferon-induced protein 44), *Gbp2* (guanylate binding protein 2). Data show mean ± standard error of the mean. Additional file 7: Table S4. Clustering of genes regulated by the gut microbiota during colonization of germ-free mice. Additional file 8: Table S5. Gene ontology categories enriched in colonization responsive gene clusters. Additional file 9: Table S6. Regulatory factors enriched in microbially regulated gene clusters. Additional file 10: Table S7. List of primer sequences used in quantitative PCR analysis.

## Abbreviations

ChIP: chromatin immunoprecipitation
CR: conventionally raised
d: days post-colonization
GF: germ-free
GO: gene ontology
HDAC: histone deacetylase
IEC: intestinal epithelial cell
LCM: laser capture microdissection
PBS: phosphate-buffered saline
