# Supplementary figures and images for: Site-specific programming of the host epithelial transcriptome by the gut microbiota
Source: Genome Biol. 2015 Mar 28;16(1):62. doi: 10.1186/s13059-015-0614-4 (PMC4404278; doi:10.1186/s13059-015-0614-4)

## Ileum

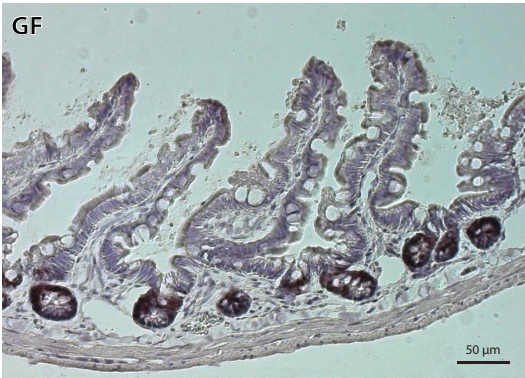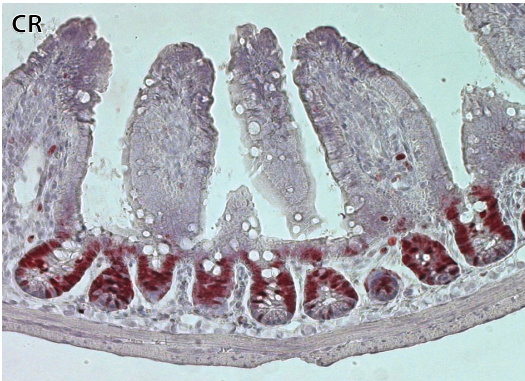

## Colon

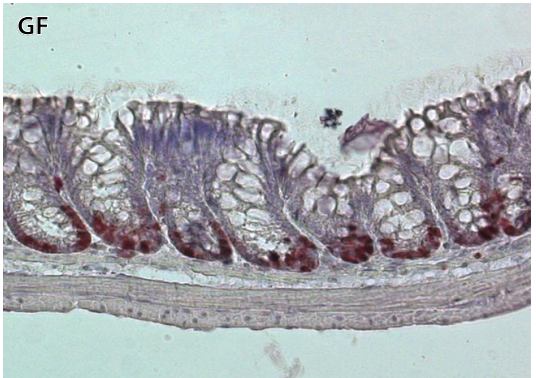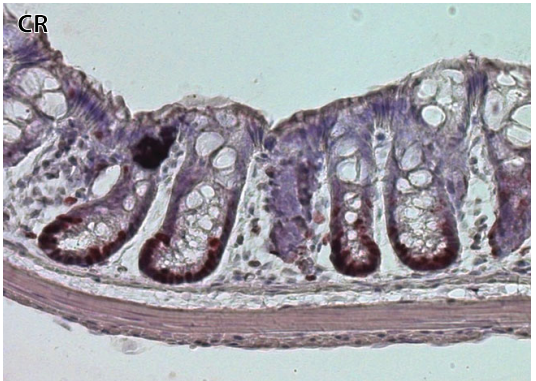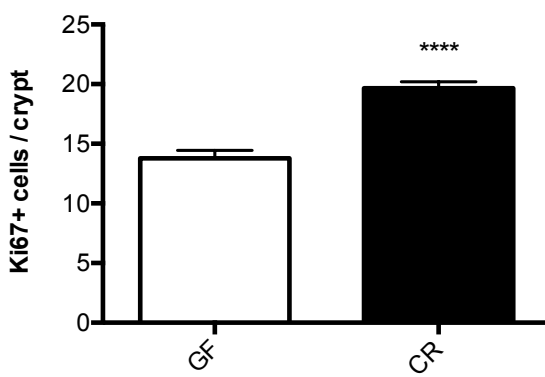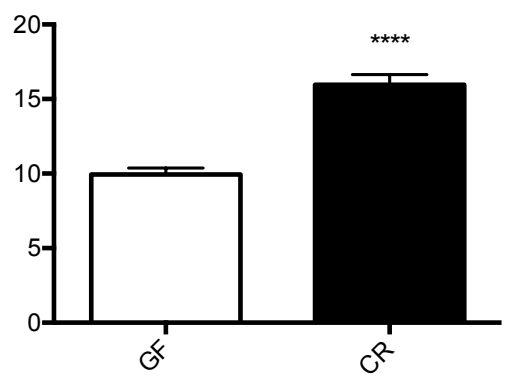

Fig. S1  
Sommer et al., 2015

Supplement: Additional file 3: Figure S1. — The microbiota induces IEC proliferation in ileal and colonic crypts. Ileal and colonic sections were stained for the proliferation marker Ki-67 and positive cells counted in the crypts. Data show mean ± standard error of the mean. ****P < 0.0001 (Student t’s test). [file 13059_2015_614_MOESM3_ESM.pdf]

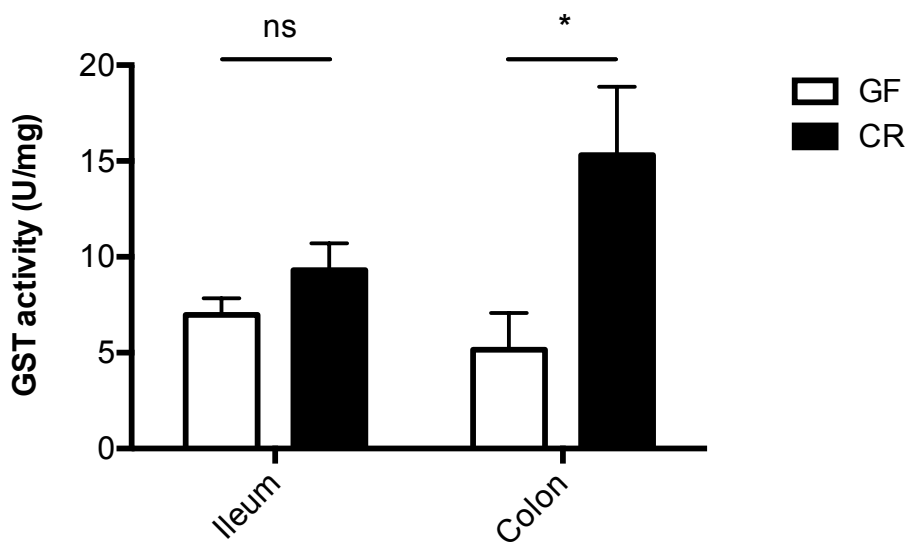

Fig. S2  
Sommer et al., 2015

Supplement: Additional file 4: Figure S2. — Glutathione-S-transferase activity is induced by the microbiota specifically in the tip epithelium in colon but not in ileum. Tip epithelium was isolated from ileum and colon of GF and CR mice and glutathione-S-transferase activity measured. Activity was normalized per milligram total protein. Data show mean ± standard error of the mean. *P < 0.05 (two-way ANOVA). [file 13059_2015_614_MOESM4_ESM.pdf]

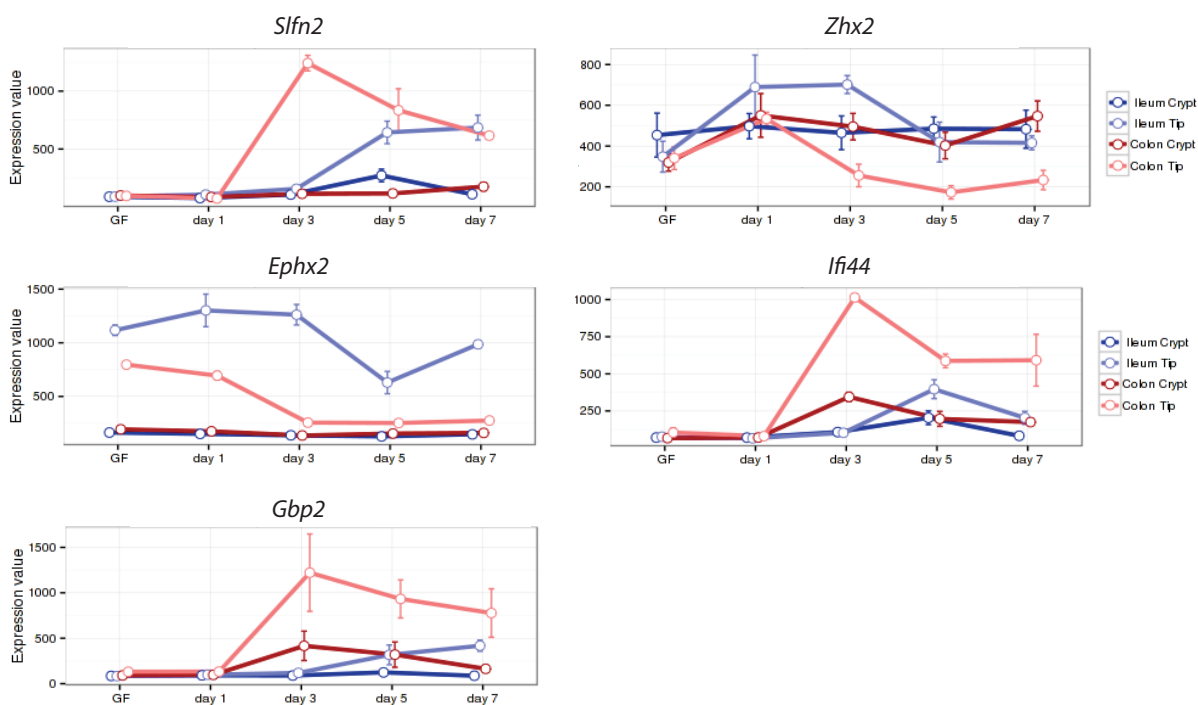

Fig. S3  
Sommer et al., 2015

Supplement: Additional file 6: Figure S3. — In colon a proportion of genes respond faster to microbial colonization than in ileum. Candidate gene expression in ileum crypt/tip and colon crypt/tip during colonization of GF mice with a normal microbiota. Slfn2 (schlafen 2), Zhx2 (zinc fingers and homeoboxes 2), Ephx2 (epoxide hydrolase 2), Ifi44 (interferon-induced protein 44), Gbp2 (guanylate binding protein 2). Data show mean ± standard error of the mean. [file 13059_2015_614_MOESM6_ESM.pdf]
